# Noli Timere: The Role of Reassuring Adults in Dealing with COVID-19 Anxiety in Pediatric Age

**DOI:** 10.3390/pediatric13010003

**Published:** 2021-01-01

**Authors:** Daniela Smirni

**Affiliations:** Department of Psychology, Educational Science and Human Movement, University of Palermo, 90128 Palermo, Italy; daniela.smirni@unipa.it

**Keywords:** COVID-19 pandemic, virus’ transmission, fear of contagion, pediatric population, state-anxiety, reassuring adult

## Abstract

Since the earliest stages of the Corona Virus Disease-19 (COVID-19) spread, the elderly has been identified as the most vulnerable and health authorities have rightly focused on that population. Minor attention was paid to pediatric populations and their emotional reactions. Actually, children and adolescents faced severe anxiety, fear and stress conditions. An efficient management of the pandemic, therefore, must take into account the pediatric population which cannot be neglected as a minor matter compared to the elderly, the economy and health care. Since the lockdown time is over, children and adolescents must recover sociality, return to living in the open air, rediscover playing, free time, aiming for the beauty of their everyday life. In order to mitigate the long-term impact of COVID-19, the key response is the reassuring presence of the adult as ‘a secure base’. The current study aimed to collect an overview of the recent references that report evidence on the role of adults in containing pandemic anxiety COVID-19 in pediatric populations, suggesting the need to ensure a reassuring presence of the adult, an effective child-parent communication, a child-friendly day and a long-lasting shared time with parents.

## 1. Introduction

In a recent study [1] unexpected high level of ‘state-anxiety’ was recorded in a group of Italian healthy older adolescents during the most restrictive period of the Corona Virus Disease-19 (COVID-19) pandemic. Surprisingly, the highest anxiety symptoms were breathing difficulties. Therefore, since the COVID-19 mainly affects respiratory functions and the sample consisted of healthy non-clinical subjects, and the assessment tools measured state and non-trait anxiety, the observed high level of anxiety was associated with a temporary feeling of apprehension that favors an increase in anxiety responses, as during the period of spread of COVID-19.

Over the past twenty years, a large body of literature had widely documented emotional reactions in children who experienced pandemic and subsequent disease-containment measures (e.g., the Severe Acute Respiratory Syndrome (SARS) outbreak; Hemagglutinin Type 1 and Neuraminidase Type 1 (H1N1- Avian Influenza A) strain; Ebola virus) [2,3,4,5,6,7].

Taken together, several recent studies from China reported that the COVID-19 pandemic can worsen existing mental health problems, and destabilize emotionally fragile children and adolescents [8,9,10] especially in the emotional dimension [2,8,11,12,13,14,15,16,17] Moreover, it is very likely that it should have long-term psychological effects, even when everyone’s life is back to normal [11,12].

Children and adolescents suddenly have to face an unknown and incomprehensible faceless enemy, which force them to distance themselves from their peers [12] to drastically change their daily reassuring habits, and to lose all certainty for their future and loved ones. Moreover, they experience the emotions, insecurity, fear and worries of adults, although the younger they are, the less they are able to cognitively understand what is happening around them. However, they feel emotionally that something very important is happening and that many behaviors around them are inexplicably changing [18,19,20]. Probably, widespread anxiety, uncertainties of parents and family [21] fear of being infected, long-term home isolation, and forced removal from schoolmates and relatives have supported an increase in anxious responses [22].

However, from the earliest stages of the spread of COVID-19, the elderly has been identified as the most vulnerable people at risk of contagion and fatal complications. Consequently, health authorities around the world have rightly focused on such frailest population. Currently, the debate on the effects of the pandemic and restrictive measures are still focused on the elderly population, the general health organization and the economic productivity. 

Minor attention was paid to pediatric populations and their emotional reactions, and despite children and adolescents faced severe anxiety, fear and stress, become a neglected minority.

On these assumptions, pandemic planning must take into account specific strategies to address the behavioral and emotional reactions of the pediatric populations, ensuring that they do not experience long-term trauma from either the pandemic disease or public health response strategies.

## 2. Aims and Methods

The current narrative review aimed to report the scientific literature evidence focused on emotional reactions to pandemic in the pediatric population and on the role of adults in containing COVID-19 pandemic anxiety. Such a review, according to recommendations by World Health Organization [23] may be of the highest importance to inform health authorities in order to plan measures, including rapid guidelines, to contain and mitigate the impact of the pandemic on children’s mental health and adolescents. 

To this end, around a hundred articles regarding recent epidemics have been reviewed following the search criteria by keywords such as: management of anxiety during epidemics; psychological and behavioral problems during epidemic stress; anxiety disorders in COVID-19 time; pandemic anxiety; pediatric populations state-anxiety; child-parent communication; parental stress management. Among these, about forty recent articles on the COVID-19 pandemic have been further investigated, because they were specific to the pediatric age and related to the current pandemic. References were identified through electronic database searching in Ovid MEDLINE, Embase, PsycINFO, Scopus and Web of Science. 

Studies inclusion criteria were as follows: (1) empirical studies and reviews; (2) written in English; (3) data on psychological factors collected during epidemics and in COVID-19 pandemic; (4) sample < 18 years of age; (5) sample of parents of children/adolescent < 18 years of age; (6) data on the prevalence of anxiety symptoms and/or factors associated with anxiety and fear management; (7) studies focused on management and possible interventions to contain children’s anxieties, to help them to express themselves and communicate their experiences from the epidemic; and (8) studies that showed dealing with the effects of COVID-19 stress.

**Figure 1:**
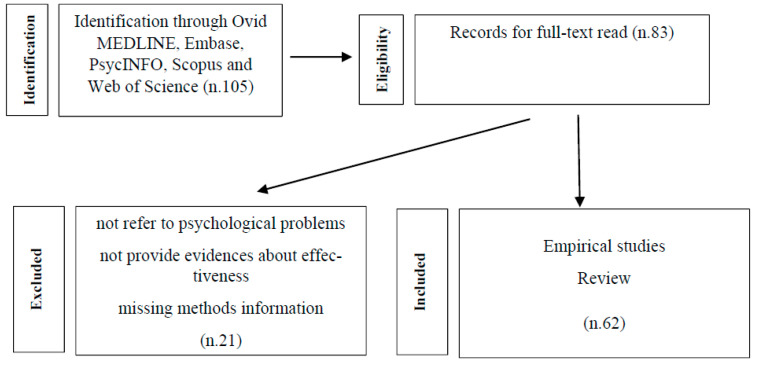
Selection of studies flowchart.

21 articles were excluded because they not refer to key topics directly, nor to health emergencies, or if full-text copies could not be obtained. General risk communication materials, such as pamphlets, posters, and infographics, were excluded as they do not provide evidences about their effectiveness. Lack of transparency due to missing methodology information was also grounds for exclusion. Figure 1 shows the selection of studies flowchart.

The final database search was run on September 2020.

## 3. Results

After an extensive literature search, twelve studies have been thorough. Table 1 and Table 2 schematically summarize the observations shown by these studies: Table 1 shows the studies that investigated the psychological impact on children and adolescents during the pandemics predating COVID-19; Table 2 shows the studies that investigated the psychological impact on children and adolescents during COVID-19 pandemic.

## 4. Psychological Impact on Children and Adolescents in Pre-COVID-19 Time

The literature of epidemics, in pre-COVID-19 time, has already documented the psychological dimension in epidemics and, namely, the emotional reactions of children and adolescents and the protective factors that can mitigate the psychological impact of the epidemic and containment measures such as social isolation.

A cross-sectional study [2] investigated post-traumatic stress reactions to pandemic and disease-containments in children and parents in areas (USA and Canada) severely affected by H1N1 or SARS. Using a mixed method approach (survey, focus groups, and interviews), data from a group of 398 parents were collected. Isolated children (about 30%) and their parents (about 25%) met criteria for post-traumatic stress disorder (PTSD). Post-traumatic stress scores were four times higher in isolated children than in not quarantined, while 28% of parents quarantined reported sufficient symptoms of a trauma-related mental health disorder, compared with 6% of parents who were not quarantined. 

Very interesting, a strong relationship was found between PTSD symptoms in parents and children. Almost 86% of parents who met the clinical symptoms for PTSD had children who also met the clinical cut-off score. Moreover, the PTSD was confirmed by public health services, in more than two-thirds of subjects. Such evidence suggested that pandemic disasters and disease-containments are affecting the lives of children and their families also socially and psychologically. Therefore, public health programs should consider the PTSD in parents and children to minimize the risk of adverse biopsychosocial consequences. 

A review, conducted by a multidisciplinary professional team [7] provides a conceptual overview of the role of fear-related behaviors and their potential impacts on epidemiologic outcomes during the 2013–2016 West Africa Ebola virus disease outbreak. The authors showed that fear and related behaviors can amplify the psychological impact of the epidemic and reduce the ability to cope with it. The invisible viral agent can create anxious uncertainty regarding risk, exposure, and infection that can lead to intense fear and dread. Consequently, fear and fear-related behaviors may play an important role in the spread of the epidemic, in the use of life-saving therapeutic measures and in increasing the risk for psychopathological behaviors. Therefore, the authors pointed out the role of effective communication to inform without spreading panic and of psychological support, especially for the emotionally fragile subjects.

Three studies underline the emotional effects of the separation of children and parents which, although may be inevitable as a protective measure of the infection, may result in depressive reactions in children, parents and even health workers [24,25] The studies suggest to treat child as a person in relation to the family as the primary source of strength and support [26].

In a wide programme of research, Koller et al. [24,25] examined, according to an ethnographic qualitative approach, the experiences of a sample of children affected by SARS, their parents, and pediatric health care providers, within a Canadian pediatric hospital. 

Using an in-depth interview method, all participants were asked to evaluate their experience, the way they deal with the pandemic and the infection control measures, and to provide suggestions for future outbreaks. Moreover, children and parents were asked specific questions about hospitalization and separation, while health care professionals were asked to describe work-related experiences while caring for SARS patients and the impact on their personal lives.

Both children, parents, and health care workers expressed feelings of emotional distress, sadness, loneliness, worry, fear and helplessness. The prevailing theme was the psychological impact of separation and isolation. Several health care providers expressed the emotional impact they felt by observing children who were separated from their families. Other recurring themes were communication difficulties due to isolation, the use of protective masks and clothing, and the limitation of family visits together with the loss of parental control and the changes in parental and professional roles. Parents expressed discomfort for the impossibility to care for their child, while the care workers covered parental role for children. 

The children’s narratives centered on four aspects, namely, increased attention to emotional reactions, collective responsibility for infection control, more effective communications and, finally, proper resources management. Additionally, the children stressed the importance of having consistent healthcare providers and allowing for therapeutic interventions that included supportive discussions and opportunities for normal play and activity. Based on these findings, the authors recommend improving patient participation by sharing information, recognizing children’s emotional reactions, providing parents with regular information about their child’s condition, and enabling them to contribute to decision making.

Nicholas et al. [26] address, in a pediatric perspective, health policy and practice implications resulting from SARS impacted on the health care facilities in Canada.

A series of semi-structured, descriptive qualitative interviews were conducted, two months after hospitalization, to examine the experiences, impacts, and implications of epidemic outbreak related health care policies for 23 participants: pediatric SARS patients, their parents, and frontline pediatric health care providers. Interview questions invited participants to identify their experiences, perceived policy and practice implications of SARS, and lessons for future outbreak.

Participants highlighted some key issues, including the development of communication strategies; releasing the vulnerability among all the subjects involved; and the development of practical guidelines. Therefore, planning strategies in pediatrics should include not only rapid containment of viruses, but also continuous and coordinated communication, and humane and accessible care.

Three very exciting studies document two different therapeutic approaches to mitigate the psychological effects of the epidemic: a support therapeutic group [16] and an expressive arts program [27,28].

In line with these observations, there is also the evidence of an observational study on a little group of SARS home-quarantined college students in Taiwan [16]. The authors described an experience with a support group lasted five sessions for a total of 500 min that included lectures, group tasks and activities, handouts, here-and-now interaction, and discussions. At an early stage, different structured relational activities were used as ice-breaking activities and to promote mutual knowledge. Subsequently, information was provided on the SARS pandemic and containment measures, and finally a structured activity was proposed in which everyone had to present ‘the peaks and valleys’ and name the main events of their life. In the second stage, the discussion centered on the concerns of the participants. They were invited to describe one’s life events that expressed different emotions (happiness, sadness, anger, fear, shame, helplessness). Many students initially pointed out that they did not like to talk about their feelings. On the contrary, they admitted that they no longer had strong feelings and that they felt rather bored when talking about the isolation. Indeed, such reduced emotional resonance profoundly impacted on their joie de vivre, their relationship life and their projection into the future. However, gradually all the members of the group described their personal experiences showing growing awareness and management of emotions. In the final stage, the participants were asked to write positive thoughts, hopes and blessings for all other participants. The thoughts were read and discussed into the group.

The authors concluded that the support group proved a powerful way for students to connect when they felt most vulnerable. Each could process their own experience by sharing it with others and by accepting the emotions and similar experiences of others. In addition, the ability to receive direct information on updated SARS projects has reduced early anxiety and developed a new perspective to fully engage in campus social activities.

Decosimo et al. [27,28] analyse the efficacy of a therapeutic psychosocial expressive arts program (‘playing to live’) focused on the growing psychosocial and mental health needs of children from three countries in West Africa who experienced a large Ebola epidemic. 

Two groups of a total of 870 children and adolescents (aged 3–18 years) who were Ebola-survivors were enrolled in an expressive arts program ‘playing to live’ for a 5-month or 3 months with the goal of supporting reintegration and decreasing stigma. The program’s framework was that expressive arts provide for children a safe space to express themselves and communicate their experiences from the epidemic, learning trauma coping skills, exploring relationships and emotions [29] and giving meanings to their chaotic environment, confusion and fear through creativity, mentorship, and peer support. 

The activities instructed children to use art, play, and storytelling to explore what they want, for example, for their future, and to help them to identify positivity within their current situation and build hope and future goals [27].

Results indicated that both treatment groups reached significant responses for the decrease of psychological stress symptoms, suggesting the urgent need for psychosocial support programming after a trauma. According to the authors, children who have access to psychosocial support and resources after trauma have a higher potential for recovery and resiliency [30] and reducing trauma stress symptoms [31].

## 5. Psychological Impact on Children and Adolescents during COVID-19

Studies on the emotional dimension and protective factors of children and adolescents in the period of COVID-19 are still quite sparse, also due to the methodological limitations that do not allow adequate control groups, comparative baseline data and longitudinal research designs [32].

A previously cited study [1] investigated state anxiety and emotion awareness in a healthy Italian sample of older adolescents during the pandemic lockdown, using the Self-rating Anxiety Scale (SAS) and the Italian Emotion Awareness Questionnaire. Over half of the SAS individual items reached a high anxiety score, and consequently the SAS total score reached an unusually high anxiety score. Analysing the single items, the item recording the highest score was item of breathing difficulties. Likewise, items referring to sleep disorder, anxiety, panic and a negative expectation of the future reached high average scores. Since the sample was a healthy, non-clinical one and the SAS measured state and non-trait anxiety, the unusually high anxiety scores observed would not appear to be attributed to the sample’s stable emotional functioning, but it is likely to be due to a temporary condition or feeling of tension and apprehension that favors a leavening of anxious responses. 

These findings supported the hypothesis that the COVID-19 pandemic may be a risk condition for an increased state-anxiety in older adolescents and suggested the need to provide 1. an effective, empathic communication system with the direct participation of older adolescents, 2. a psychological counselling service for the stress management of adolescents.

Four different studies from China examined large groups of children and adolescents and found significant levels of anxiety and depression or somatic disorders (bodily aches, pains or difficulties breathing) in response to the COVID-19 pandemic [8,11,13,14].

A cross-sectional survey [33] explored the impact of the COVID-19 pandemic on somatic symptoms and concerns about pandemic in a sample of Chinese college students (n = 198) and of primary school students (n = 209), using the Somatic Self-rating Scale and a questionnaire aimed at examining three different apprehensions related to the Covid-19: daily needs, effectiveness of prevention and control measures, the threat to life and health for both the participants and their families.

Concerns about the outbreak were associated to somatic complaints in both groups, although the psychological impact was different. Primary school children were mainly concerned about the threat to life and health, and this concern was associated with anxiety and somatic symptoms. However, they had a lower incidence of somatic symptoms than college students (2.39% vs. 34.85%). College students were concerned for all the three domains, and all three of the concerns rated were linked to anxiety and depression and to somatic complaints. The authors argued that the differences between groups could be related to the fact that primary school students are still protected by parents who attend to their daily needs and control measures, while the majority of college students are independent and, as result, they tended to worry about all aspects of the pandemic and exhibit more somatic symptoms than primary school children. However, it is interesting to note that college students in the pandemic condition appear emotionally fragile, despite the independence they have achieved from their parents.

Therefore, data support the need to differentiate psychological measures in relation to different ages. Specifically, the authors pointed out that psychological health of children should begin with an appropriate health education for parents to protect their children from psychological distress [34]. Similarly, for college students, the authors emphasize the importance of direct health education aimed at improving knowledge of COVID-19 to promote prevention and control measures [35,36].

Xie et al., [13] in a cross-sectional study, investigated depressive and anxiety symptoms, by the Children’s Depression Inventory–Short Form and the Screen for Child Anxiety Related Emotional Disorders, among a large group of primary school children (2330) in home confinement during the COVID-19 in a Province of China.

Overall, a percentage of students higher than other investigations in primary schools of China [37] reported depressive (22.6%) and anxiety symptoms (18.9%). 

Interestingly, the levels of anxiety and depression were influenced by positive or negative attitudes towards the epidemic. Students who were slightly or less concerned about being affected by COVID-19 showed lower anxiety scores and lower depressive symptoms. Conversely, students who were not optimistic about the outbreak they showed higher anxiety scores and depressive symptoms. Similarly, anxiety and fear of contagion and feelings of life threat were higher in highly infected areas. No significant association was found between demographic characteristics and anxiety symptoms. Sex did not predict anxiety and depression. According to the authors, the increase in children’s depressive symptoms must be associated with the reduction of outdoor activities and social relationships. 

In a cross-sectional study, Zhou et al. [14] using an online survey, assessed a very large number of students (8079), aged 12–18 years. Socio-demographic information and students’ awareness of COVID-19 were collected. Participants were asked about their familiarity with COVID-19 prevention and control, whether they had taken all prevention and control measures to avoid contagion, and about their opinions towards the COVID-19 trend projections. Depressive and Anxiety symptoms were assessed by the Patient Health Questionnaire [38] and the Chinese version of the Generalized Anxiety Disorder scale [39].

Overall, the prevalence of depressive and anxiety symptoms was 43.7% and 37.4%, respectively. Age was significant, the higher the grade, the greater the prevalence of depressive and anxiety symptoms. Senior high school was a risk factor, confirming Liu’s data [11] documenting greater emotional fragility in college students compared to primary school students. Sex also was significant, as girls showed a higher level of anxiety and depression than boys. Similarly, anxiety and depressive symptoms in rural areas were significantly higher than in cities. This was consistent with previous studies, which found nearly twice as much emotional disorders among the poor as the rich [40,41]. Interestingly, the level of knowledge and awareness about COVID-19 and positive attitude towards the development of epidemic appeared as protective factors against depressive and anxiety symptoms. This was consistent with previous studies showing that wearing a mask and practicing hand hygiene reduces the level of anxiety and depression [42]. According to the authors, provide accurate information, such as the progress of medicines and vaccines, and refute alarming news can reduce anxiety levels, and favor a better emotional stability [42].

Age-related differences have been documented by a cross-sectional study conducted by Jiao et al., [8] among 320 children and adolescents, aged 3–18 years, under protective isolation, using an online questionnaire, completed by the parents. Clinging, inattention, and irritability were the most severe symptoms showed by the sample in all age. The younger group (3–6 years) was more likely than older to manifest symptoms, such as clinginess and fear that family members could be infected, while children aged 6 to 18 years were more likely to show inattention and persistent inquiry. Moreover, the study confirmed Xie’s data [13] that fear and anxiety were higher in children residing in highly epidemic areas.

Based on the data analysis, the authors pointed out that resilience should be implemented in children and teens. Parents may support the child by increasing communication to address their fears and concerns, playing collaborative games to alleviate loneliness, encouraging physical activity, and using music, media entertainment, reading, and singing to reduce the worry, fear, and stress [43]. Moreover, the authors suggest to pay attention to sleep difficulties and nightmares, prevent increased daytime sleep and model a positive psychological attitude to reduce stress, and focus on positive activities. 

## 6. Discussion 

The focus of the current overview has been to examine research studies, over the past two decades, reporting on the effects of COVID-19 and previous epidemic events on emotional responses in the pediatric population and on the role of adults in containing COVID-19 pandemic anxiety.

Overall, the reviewed studies, both in the pre-COVID-19 and in the COVID-19 time, all agree in supporting the devastating action of epidemic events and containments measures on psychological well-being and mental health of childhood and adolescence [32,44,45]. Moreover, the studies broadly showed that parents’ reassurance behaviours and social support may mitigate the negative effects of epidemics on stress response in developmental age [46,47,48].

The recurrent themes in pre-COVID studies were the isolation, social separation and the loss of reassuring communication and information. In all studies, prolonged separation from significant adults has been for children and adolescents a very serious experience of loss and rejection, resulting in mourning depressive reactions, anxiety and fear. The communication between children, relatives, peers, friends and parents became extremely complex. Isolated children and parents achieved significantly higher post-traumatic stress levels than children or parents not in isolation [2].

Probably, as evidenced by a multidisciplinary overview [7] fear and fear-related behaviors could amplify the psychological impact of the epidemic and support a sort of circularity of anxiety, reducing the ability to cope with it. Therefore, fear and fear-related behaviors may play a role in increasing the risk for psychopathological behaviors. The authors pointed out the main role of effective communication to inform without spreading panic.

Interestingly, research reported that most parents reaching significant levels of post-traumatic stress have children with high levels of stress and vice versa [2].

The limitation of visits or even their complete prohibition increased the experience of loneliness and helplessness [24,25,26]. It almost seems that the forced separation due to the epidemic undermines the child’s attachment to the secure figure of the parent. The psychological literature fully agrees on the key role of attachment to the parental figure for the emotional and social growth of the child and on the confusion resulting from the loss of this bond. Even the relationship with an unknown health worker proposes an experience of relational stress that may refer to the paradigm of the strange situation [49,50,51].

Healthcare providers also expressed feelings of helplessness and inadequacy in the face of the loneliness of their patients, unable to replace the parents, although they are almost engaged with a parental role. Moreover, they cannot ensure a consistent presence as they alternate according to mobile shifts, and they circulate protected by a mask and clothing that give them a not very familiar reassuring aspect. Parents, in turn, felt frustrated at not being able to exercise their parental role and not being able to actively participate in decision-making. All the protagonists, therefore, experience and complain of a persistent condition of loneliness and helplessness in the face of a mandatory and protective isolation to contain the contagion.

Studies documenting therapeutic interventions to alleviate the psychological impact of the pandemic describe activities just focused on recovering and improving communication. Within psychological support groups [16] or expressive arts groups [27,28], students were invited to verbalize and reframe their experiences of fear, anxiety and depression together with their peers and qualified professionals. The therapeutic expressive or support group acts precisely on the recovery of a more effective communication and everyone can share with peers fears and stressful experiences and feel the support of sharing from those who have experienced similar emotions.

Strangely, in the psychological support group, many home-quarantined students, when talking about the SARS quarantine, in the early stages, manifested some sort of underlying emotional detachment and a widely emotional disengagement from events and people. However, under the therapeutic prompting of the leader, thereafter some began to tell their experience showing that, within the group, communication was becoming therapeutic by confronting others’ similar emotions and experiences.

Likewise, the psychosocial expressive arts programs ‘Playing to live’ developed in Liberia to promote play and expression as a measure of healing after Ebola Virus Disease showed that children found enjoyment outside of art and play, increasing their hope and self-esteem, being with peers and receiving support from adults. A new, artistic unexplored communication modality makes children aware of their creative strength, and begins their individual’s emotional and cognitive recovery [27,28]. The studies, therefore, show that improving communication is an effective and powerful way to reframe emotions of the pandemic and process them with a greater realistic view [16].

A relevant topic in all studies was the matter of information to reduce anxiety and realistically review mistaken beliefs and fears. Both children and adolescents feel reassured to receive up-to-date information on the epidemic when the information is accompanied by a reassuring attitude about protective containment measures and progress by scientists who are studying the disease and possible treatments.

Studies on the psychological effects of COVID-19 in pediatric age are relatively fewer than those reported in previous epidemics. However, the studies, from different researchers and from different countries, confirmed the findings of the pre-COVID-19 searches on the impact of the current epidemic on the emotional life and behavior of children and adolescents.

An emerging finding concerns the different response in different ages. Older adolescents appeared as more sensitive to anxiety than younger children and more fragile and vulnerable in the face of the new and complex problems of protection against the spread of the virus [11]. Probably, according to researchers, the younger child can still rely on the protection and care of adults, while the older adolescent feels exposed in the first person, less protected from parents [11]. Moreover, according to some studies, younger children (3–6 years) manifest their discomfort with childlike regressive behaviors of excessive attachment and expressing the fear of losing their parents for the infection, while older children (6–18 years) show more attention difficulties and persistently asking reassurance questions. All this would imply, according to the researchers, that the child’s anxieties should be managed involving mainly the parents through parent training, while on older adolescents direct therapeutic treatments could be implemented [8].

It is interesting to note that the studies showed a significant relationship between the attitude towards the pandemic, the level of knowledge and awareness of COVID-19 and the level of anxiety and depression. Students who were very worried about being infected had higher anxiety and depression scores, while, by contrast, students less concerned about the infection had lower scores [14]. Similarly, those who routinely wore masks and regularly practiced hand hygiene had lower levels of anxiety and depression. In other words, a valid information, a realistic approach to the severity of the pandemic and an implementation of the available containment strategies appear as protective factors for anxiety control. In the same view, anxiety levels were higher in rural or low-wealth areas. Probably, in such contexts, a lower ability to manage information in a critical way and to protect oneself from false or contradictory news favors a greater rise in anxiety [40,41].

Therefore, taken together, the studies reviewed, show that the pandemic is a catastrophic event for the general health of the population, for the economics and for the mental health of the youngest subjects and more developmentally fragile.

Any outbreak, according to the literature, appears as an uncontrollable, unpredictable and cumulative stress factor occurring outside the control of everyone, including the parents, and increasing fear, anxiety, uncertainty, and feelings of helplessness on the everyday life of children and parents. Therefore, pandemic and restrictive measures, in line with the learned helplessness stress model [52], may be viewed as an important cumulative stress source, in which a faceless and invisible enemy require full and persistent alert. In pediatric age, emergencies, as chronic and traumatic stress, may be severely dangerous because they stimulate an excessive release of stress hormone cortisol [53,54], by the limbic-hypothalamic-anterior pituitary-adrenal cortex system [55,56], and, over time, according to the cumulative stress hypothesis [57], they may reduce long-term health and increase the vulnerability to stress-related pathologies [58,59]. Moreover, dysregulation of stress hormones could have lasting effects on cognitive processes [46,59,60,61,62], given that the hippocampus, with its high density of glucocorticoid receptors, appears as a structure particularly involved in the negative feedback loop for the regulation of cortisol [63,64] and in cumulative exposure to elevated cortisol levels [65]. Furthermore, high cortisol, corticotropin and epinephrine can affect the sensitivity of fear responses mediated by the amygdala and alter the hormonal response to social challenges, increasing the risk of immune dysfunction and other health problems [66,67].

In sum, according to the reviewed literature, children and adolescents are not indifferent to the dramatic impact of the COVID-19 epidemic, while an efficient management of the pandemic must take into account the pediatric population which cannot be neglected as a minor matter compared to the elderly, the economy and general health care [4]. Since the lockdown time is over, children and adolescents must recover sociality, return to living in the open air, rediscover playing, free time, aiming for the beauty of their everyday life, without forgetting basic rules for keeping COVID-19 away.

Therefore, in line with literature’s data, the core values to prevent or mitigate the impact of COVID-19 and containment strategies must be focused on the verbal and nonverbal reassuring presence of the adult as an important protective factor. Specifically, planning to deal with the effects of pandemic stress should ensure a reassuring presence of the adult, an effective child-parent communication, a child-friendly day, and a long-lasting shared time with parents. 

## 7. Ensure Reassuring Presence of the Parents 

Researchers in child development widely described the negative impact of separation from parents on hospitalized children. Bowlby and Robertson [49,50,51] associated intense separation anxiety with poor attachment between parent and child. According to Bowlby and Ainsworth’s secure base theory, children must strongly fee the reassuring presence of the parents as ‘secure base’ [51]. Child or adolescent must feel they can always come back on a secure island where someone, strong and experienced, is ready to welcome him and to comfort and reassure him to face the world adequately. 

The study by Liu et al. [11] documented that college students, in a pandemic condition, still appear quite emotionally fragile even if they have reached a level of autonomy and independence from adults. The study showed a greater presence of somatic symptoms in college students than in primary school children (34.85% vs. 2.39) and a wider range of concerns regarding the epidemic linked to anxiety and depression.

Moreover, research on dealing with stressful situations has broadly showed the effectiveness for children of social support, especially from significant adults, to markedly reduce the psychobiological responses to stress [68]. Some study found that parents with higher levels of anxiety and distress have reported a higher level of distress in their children [21].

Therefore, the first and most effective measure is to be empathically present and convey calm with the entire repertoire of verbal and non-verbal communication. Particular attention should be paid to children with neurodevelopmental disorders because they could have communication difficulties about their feelings and sensation of pain [69,70,71].

Anxiety and stress may be much more contagious than any virus, and parents are the most important example for the child [18]. To convey calm, the key factor is feeling calm, to take care of yourself, to live all-around and to do any activity that makes feel good. In anxiety and distress conditions, children learn from adults how to manage stress [20].

Moreover, children need to feel that their anxieties and fears are understood and shared by significant adults. According to the inhibition theory [72], when you can’t talk about traumatic experiences for a long time, it becomes cumulative stress that makes you more vulnerable to stress-related diseases. 

## 8. Ensure Effective Child-Parent Communication

The studies examined in the current review document that the level of knowledge and awareness about COVID-19 may be a protective factor [14]. However, it is important to note that any information can have a different value in relation to the credibility of the person who provides it. Sometimes, in pre-COVID pandemics, official recommendations have been met with skepticism [73]. Washing your hands, coughing under a mask or at the elbow, keeping social distances, cleaning surfaces, not touching your face can all be simple behaviors to adopt. However, they become acceptable if the proposer is as authoritative for the child as his parent can be. Therefore, an effective and honest communication child-parents supports a proper understanding of what is going on and stimulates the child to safely express their feelings [74]. Children of any age, even the youngest, feel the emotional states around them [19] and will get even more anxious if they don’t perceive the adults as sincere and easy to understand. Unexplained or uncertain behaviours may be felt as a threat and as anxiety source.

Moreover, according to the magical thinking construct [75], children, because of confusion about mind and reality, attempt to make sense on their own [76] and they may belief that their thoughts or behaviors have a causal effect on unrelated real events, such as disease. Therefore, providing children with an accurate meaningful explanation [77], will ensure they do not feel unnecessarily frightened or guilty, or inappropriately blame themselves or feeling that the illness is a punishment for previous bad behaviour and reinforce the child’s positive and preventive behaviors [78].

## 9. Ensure a Child-Friendly Day 

Containing children’s anxieties means helping them not to experience boring and uninteresting days. Inactivity may easily feed parasitic thoughts, fears, and ritualistic and perseverative ideas and behaviors [28]. On the contrary, several studies [8,27,28] pointed out that media entertainment, reading, physical exercise may be protective factors to mitigate distress resulting from pandemic condition. Being involved in stimulating activities promotes the safety, psychological well-being and mental health of children. Of course, it’s not about forcing them, without any break, into ‘demanding’ activities, but live as children in a structured, stimulating and welcoming world in which the playful-recreational dimension and commitment are integrated [28].

Moreover, even careful use of television and, more generally, of informatic tools (videogames, play stations) can be an interesting and inspiring way into a child’s day. It is important to limit exposure to media news by selecting only official information and make sure that correct messages are received from children. But it is the diligent and reassurance presence of the adult that makes the difference.

## 10. Ensure a Long-Lasting Shared Time with the Parents

The restrictions of the pandemic can be rethought as a challenge to improve relationships with children by staying close and sharing their life and their ways of expression. Not least, a climate of relaxation, mediated by significant and collaborative playful and recreational activities, becomes a favourable opportunity for the psychological well-being of the entire family, especially for more fragile people, though in adults can promote a redefinition of the forgotten everyday life relevant values.

A qualitatively significant time sharing represents a crucial response to the child’s fears and anxieties and to child’s need to feel loved and appreciated, while the lockdown experience may be a condition to disrupt this process on multiple levels [79].

## 11. Conclusions

The current narrative review focused on emotional reactions to pandemic in the pediatric population and the reassuring role of adults in dealing with pandemics. 

Pandemic and restrictive measures, in line with the learned helplessness stress model [52], may be viewed as an important cumulative stress source. The literature, in pre-COVID-19 time, has already documented the psychological dimension in epidemic events and, namely, the emotional reactions of children and adolescents. Similarly, literature data, in the COVID-19 time, consistently confirmed an increase in depressive and anxious symptoms in children and adolescents [8,11,13,14].

Several studies pointed out that social isolation, separation, and communication difficulties were the recurring issues in complaints of both children and adults. Social isolation may be felt as a serious source of anxiety by the child, by the parents and also by health professionals. Research reported that most parents reaching significant levels of post-traumatic stress have children with high levels of stress, supporting a sort of circularity process of anxiety within the group. According to a conceptual overview, fear and fear-related behaviors can amplify the psychological impact of the epidemic and reduce the ability to cope with it [7].

Conversely, therapeutic approaches focused on peer communication, within psychological support groups or expressive arts groups, have proven to be powerful tools in pediatric age to recover emotional stability after stressful experiences due to pandemics. 

Moreover, the level of knowledge and awareness about COVID-19 and positive attitude towards the development of epidemic appeared as protective factors against depressive and anxiety symptoms. Therefore, all searches emphasized the role of reassuring information and social support in preventing or mitigating psychological impact of pandemic and containment strategies [46,47,48].

Future research will have to focus, with rigorous and appropriate methodologies, on the long-term effects of the psychological impact of epidemics on the pediatric population. 

## Figures and Tables

**Table 1:** Studies on the psychological impact on children and adolescents in pre-COVID-19 time.

Study	Study Design	Participants	Sample Size	Sample Age	Measures	Results
Sprang & Silman, 2013 [2]	Cross-sectional	Parents of children in areas affected by H1N1 and SARS	398	18–67 years	-parent-report version of the University of California at Los Angeles Post Traumatic Stress Disorder Reaction Index (PTSD-RI)-PTSD Check List-Civilian Version (PCL-C).	mean post-traumatic stress scores were four times higher in children who had been confinement measures than in those who were not quarantined
Shultz et al., 2016 [7]	Review	Individuals in areas affected by Ebola	-	-	Assessment of fear-related behaviors during Ebola pandemic	fear and related behaviors can amplify the psychological impact of the epidemic and reduce the ability to cope with it
Koller et al., 2006 [24]	Cross-sectional	(a) pediatric patients affected by SARS (b) parents (c) pediatric health care providers	(a) 5 (b) 10 (c) 8 tot: 23	(a) 6–18 years	Emotional effects of separation children/parents	children, parents, and health care workers expressed feelings of emotional distress, sadness, loneliness, worry, fear and helplessness
Koller et al., 2010 [25]	Cross-sectional	Children and adolescents	21	5–19 years	interview about knowledge of SARS, experience of hospitalisation and isolation, coping mechanisms, and reintegration to home and community	psychological responses to SARS were extensive, causing social and emotional difficulties for children and adolescents
Nicholas et al., 2008 [26]	Cross-sectional	(a) pediatric patients with suspected SARS (b) parents (c) pediatric health care providers to SARS patients	(a) 5 (b) 10 (c) 8 tot: 23	(a) 5-17 years	Interview to examine the experiences, impacts, and implications of SARS-related health care policies for patients, parents, and health care providers	-development of communication strategies for responding to SARS -easing vulnerability among all stakeholders
Decosimo et al., 2017 [27] and 2019 [28]	two-treatment group design	Children and adolescents in areas affected by Ebola	870	3–18 years	therapeutic psychosocial expressive arts program	arts provide for children a safe space to express themselves and communicate their experiences from the epidemic, learning trauma coping skills, exploring relationships and emotions

Legend: H1N1 Hemagglutinin Type 1 and Neuraminidase Type 1 (Avian Influenza A); **SARS** Severe Acute Respiratory Syndrome; **PTSD** Post Traumatic Stress Disorder.

**Table 2:** Studies on the psychological impact on children and adolescents during COVID-19.

Study	Study Design	Participants	Sample Size	Sample Age	Measures	Results
Smirni et al., 2020 [1]	Cross-sectional	Healthy students	148	17–19 years	-Zung Self-Rating Anxiety Scale -Emotion Awareness Questionnaire	-high anxiety score -high emotional awareness score
Jiao et al., 2020 [8]	Cross-sectional	Healthy children and adolescents	320	3–18 years	Questionnaire on psychological and behavioral problems commonly used for a cross-cultural assessment of anxiety disorders	clinging, inattention, and irritability were the most severe symptoms
Liu et al., 2020 [28]	Cross-sectional	Primary school children	209	fifth and sixth grades, age unspecified	-Somatic Self-rating Scale -Questionnaire on apprehensions related to the Covid-19	concern about the threat to life and health, associated with anxiety and somatic symptoms
Xie et al., 2020 [13]	Cross-sectional	Primary school children	2330	from second to sixth grades, age unspecified	-Children’s Depression Inventory–Short Form -Screen for Child Anxiety Related Emotional Disorders	-high depressive symptoms -high anxiety symptoms
Zhou et al., 2020 [14]	Cross-sectional	Healthy students	8079	12–18 years	-Patient Health Questionnaire -Chinese version of the Generalized Anxiety Disorder scale	the higher the age, the greater the prevalence of depressive and anxiety symptoms

## Data Availability

Not applicable.
